# Effect of the COVID-19 Pandemic on Treatment Adherence Among Children With Congenital Adrenal Hyperplasia

**DOI:** 10.7759/cureus.27762

**Published:** 2022-08-07

**Authors:** Samahir A Alsulaimani, Ahlam Mazi, Mohammed Bawazier, Ali Bahabri, Wael Eibani, Abdulrahman Batarfi, Abdulmoein Al-Agha

**Affiliations:** 1 Pediatric Department, King Abdulaziz University, Jeddah, SAU; 2 Pediatric Department, King Abdulaziz University, Faculty of Medicine, Jeddah, SAU

**Keywords:** covid-19 pandemic and cah treatment, lockdown and cah treatment compliance, effect of covid-19 on cah, cah treatment compliance, impact of covid-19 on treatment, covid-19 and cah

## Abstract

Background: This study determined the effect of the COVID-19 pandemic on adherence to medication among children with congenital adrenal hyperplasia (CAH) living in the Kingdom of Saudi Arabia (KSA).

Methods: Data were collected from April 2020 to April 2022 through face-to-face or telephonic interviews at a virtual paediatric endocrine clinic in Jeddah, KSA.

Results: A total of 55 children, with a mean age of 12.9 ± 5.8 years, participated in the study. Most children (32/55, 58%) were administered treatment by their mother. Before the COVID-19 pandemic, 51 patients (93%) reported adhering to their CAH treatment. After the onset of the COVID-19 pandemic, this number decreased to 49 (89%, p = 0.516). The most common reasons for non-adherence before and after the pandemic included restricted access to medication (supply and financial problems) and challenges in obtaining new prescriptions and refills. These challenges increased after the onset of the pandemic. Before and after the onset of the pandemic, mothers with a university degree were significantly more likely to administer medication than mothers without a university degree, but fathers’ education level did not affect their role in medication administration, before or after the onset of the pandemic.

Conclusion: This study confirms that the COVID-19 pandemic did not have a significant effect on medication adherence in children with CAH in Jeddah.

## Introduction

Congenital adrenal hyperplasia (CAH) is a group of autosomal recessive genetic disorders characterised by a deficiency of enzymes involved in the biosynthesis of corticosteroids and mineralocorticoids secreted by the adrenal glands. The disease has a variety of phenotypes [1]. In Saudi Arabia, the incidence of CAH is associated with consanguinity and the incidence is estimated at 1 per 5000 to 1 per 7908 live births [2,3].

The 2019 outbreak of Coronavirus disease (COVID-19) in Wuhan, China, led to a pandemic that was officially proclaimed in March 2020 and continues at the time of writing (July 2022). The virus is transmitted between humans via the droplet and faecal-oral routes [4]. To prevent infection spread, lockdowns, social distancing, and other public health measures have been implemented worldwide [5].

Medication adherence among children and adolescents with chronic diseases is determined by several factors. These factors include medication accessibility and type; family and caregiver structure; healthcare system characteristics; and cultural, psychological, and socioeconomic factors [6]. The COVID-19 pandemic has affected follow-ups, prescription refills, and medication adherence rates. Medication adherence and regular follow-up for children with CAH are crucial to prevent complications from under or over-treatment of the disease [7,8].

To the best of our knowledge, this is the first study to evaluate the effect of the COVID-19 pandemic on treatment adherence among patients with CAH.

## Materials and methods

Study design and patients

A cross-sectional descriptive study was conducted at the King Abdulaziz University Hospital, Jeddah, Kingdom of Saudi Arabia (KSA). Data on child/adolescent patients with CAH who are on medication were collected from their parents or guardians or from the adolescent patients themselves in Jeddah city via face-to-face or virtual interviews from April 2020 to April 2022. In this study, 55 patients were recruited according to the following inclusion criteria: aged 0-18 years, a diagnosis of CAH, and a prescription for hydrocortisone therapy. Patients were excluded from this study if they were aged >18 years, lived outside the country, had missing medical record data, or missed clinic appointments. This study was limited to 55 patients owing to the rarity of the disease.

Variable assessments

Patient data were anonymised and stored in the principal investigator’s office. Only the study co-authors had access to the dataset. The Research and Ethics Committee of the King Abdul-Aziz University Hospital approved the study protocol (Reference No. 12-22, No. of registration at the National Committee of Biomedical and Medical Ethics HA-02-J-008). All study procedures adhered to the provisions of the Helsinki Declaration. Written consent was obtained from adolescent patients (10-18 years) and their caregivers and from the primary caregivers of paediatric patients (0-9 years and 11 months) before the clinical interview. Face-to-face interviews were conducted at a paediatric endocrine clinic during the study period for children older than 10 years; data of younger children were obtained from their caregivers to assess adherence through structured interviews. The interviews were conducted in Arabic and consisted of three parts. The first part recorded information on demographic characteristics, such as socioeconomic status, gender, age, and the people responsible for medication administration and their level of education. The second and third parts recorded medication adherence and the regularity of clinic follow-up visits and laboratory tests, respectively. Treatment-related items included medication type and frequency (hydrocortisone and fludrocortisone), adherence, and reasons for non-compliance with clinic visits before and after the onset of the COVID-19 pandemic.

Statistical analysis

The data were coded, tabulated, and analysed using SPSS version 20 (IBM Corp., Armonk, NY, USA). Categorical variables are presented as counts and percentages, and comparisons between groups were performed using the chi-square test. Continuous variables are presented as means and standard deviations. Non-normally distributed variables were tested using the Mann-Whitney U test and Kruskal-Wallis test. Correlation analysis was performed using Spearman’s rank correlation co-efficient. P-values <0.05 indicated statistical significance.

## Results

The characteristics of the patients and their parents are presented in Table 1. The mean age of the study patients was 12.9 ± 5.8 years; 22 fathers (40%) had a high school education, and 25 mothers (45%) had a university education. Most children (58%) were administered treatment by their mothers.

**Table 1:** Patient characteristics (N = 55)

Variable	No. (%)	Mean	SD
Age (years)		12.9	±5.8
Gender			
Male	17 (31)		
Female	38 (69)		
Nationality			
Saudi	27 (49)		
Non-Saudi	28 (51)		
Father’s education level			
Primary school	6 (11)		
Elementary school	10 (18)		
High school	22 (40)		
University	17 (31)		
Mother’s education level			
Primary school	5 (9)		
Elementary school	12 (22)		
High school	13 (24)		
University	25 (45)		
Patient’s education level			
Primary school	23 (42)		
Elementary school	17 (31)		
High school	8 (15)		
University	7 (13)		
The person responsible for medication administration			
Patient	15 (37)		
Mother	32 (58)		
Mother and father	8 (15)		

Before the COVID-19 pandemic, 51 patients (93%) reported adhering to their prescribed CAH treatment. However, after the onset of the COVID-19 pandemic, this number decreased to 49 (89%, p = 0.516). Reasons for non-adherence before and after the pandemic are shown in Table 2. These included restricted access to medication (supply and financial problems) and challenges in obtaining new prescriptions and refills, which increased after the onset of the COVID-19 pandemic.

**Table 2:** Reasons of non-adherence to medication among children with congenital adrenal hyperplasia before and after the onset of the COVID-19 pandemic

Variable	Before COVID-19 (N=55) (No.%)	After COVID-19 (N=55) (No.%)	p-value
Forgetfulness	3(5)	3(5)	0.991
Medication unavailability (pharmacies, financial issues)	1(2)	4(7)	<0.001
Difficulties refilling or acquiring prescription	2(4)	4(7)	<0.001
Missing or failing to book a physician appointment	2(4)	3(5)	0.071

A total of 45 patients (82%) were prescribed hydrocortisone three times per day. The adherence rates before and after the onset of the COVID-19 pandemic were comparable. In addition, the number of patients who were prescribed hydrocortisone three times a day was comparable before and after the pandemic’s onset (p = 0.999).

Before the pandemic, 41 patients (75%) underwent laboratory testing every three months. After the onset of the pandemic, this decreased to a total of 37 patients (67%). Similarly, before the pandemic, a total of 40 patients (73%) attended scheduled follow-up visits every three months. After the onset of the pandemic, this decreased to 37 patients (67%), primarily owing to the availability of virtual clinics.

Before and after the onset of the pandemic, mothers with a university degree were significantly more likely to administer medication. The father’s level of education was not associated with medication administration before or after the pandemic’s onset.

The patient characteristics associated with adherence to treatment with hydrocortisone and fludrocortisone before and after the pandemic’s onset are shown in Tables 3-4, respectively. There were no significant differences in patient adherence to the medication before and after the onset of the COVID-19 pandemic according to patient characteristics, including age, gender, and nationality.

**Table 3:** Patient characteristics and adherence to hydrocortisone treatment before and after the onset of the COVID-19 pandemic

Variable	Before COVID-19		p-value
	Adherent N=51 No. (%)	Non-adherent N=4 No. (%)	
Age(years)	12.7 ± 5.8	15.3 ± 6.2	0.943
Gender			
Male	16(94)	1(6)	0.791
Female	35(92)	3(8)	
Nationality			
Saudi	25(93)	2(7)	0.97
Non-Saudi	26(93)	2(7)	
	After COVID-19		
	Adherent N=49 No. (%)	Non-adherent N=6 No. (%)	
Age(years)	12.8 ± 5.8	13.8 ± 6.3	0.79
Gender			
Male	15 (88)	2(12)	0.892
Female	34(89)	4(11)	
Nationality			
Saudi	25(93)	2(7)	0.43
Non-Saudi	24(86)	4(14)	

**Table 4:** Patient characteristics and adherence to fludrocortisone treatment before and after the onset of the COVID-19 pandemic

Variable	Before COVID-19			p-value
	Adherent N=41 No. (%)	Non-adherent (N=2) No. (%)	NA (N=12) No. (%)	
Age(years)	12.8 ± 5.7	12.0 ± 1.4	11.7 ± 5.9	0.123
Gender				
Male	13(76)	1 (6)	3(18)	0.76
Female	28(74)	1(3)	9(24)	
Nationality				
Saudi	22(85)	1(4)	4(12)	0.464
Non-Saudi	19(68)	1(4)	8(29)	
	After COVID-19			
	Adherent (N=41) No. (%)	Non-adherent (N=3) No. (%)	NA (N=11) No. (%)	
Age(years)	12.8 ± 5.7	12.0 ± 3.6	11.5±6.2	0.305
Gender				
Male	13(76)	1(6)	3(18)	0.957
Female	28(74)	2(5)	8(21)	
Nationality				
Saudi	22(85)	1(4)	4(15)	0.508
Non-Saudi	19(68)	2(7)	7(25)	

## Discussion

The COVID-19 pandemic has affected different aspects of the lives of children and adolescents with CAH, including but not limited to medication adherence and availability and follow-up in the clinic. This study assessed the effect of the COVID-19 pandemic on medication adherence in patients with CAH and showed that there was no significant difference in medication adherence before and after the onset of the COVID-19 pandemic. Several factors affect young people’s treatment compliance, including caregiver structure, treatment type, administration frequency, follow-up appointment scheduling, and medication sources.

Several studies have been conducted on adults and children to assess medication adherence in Saudi Arabia. A study of adult diabetes patients showed reduced adherence to medication and a healthy lifestyle during the COVID-19 pandemic. Adherence was assessed by a questionnaire [9]. Another questionnaire-based study of children with type-1 diabetes mellites showed that adherence to medication increased during the COVID-19 pandemic [10].

Early diagnosis and treatment of CAH helps prevent poor outcomes. Treatment includes glucocorticoid replacement in the form of hydrocortisone at a dose of 10-15 mg/m2/day three times per day and mineralocorticoid replacement with 9α-fludrocortisone acetate at a dose of 100 µg once or twice daily. Sodium chloride replacement is recommended for infants [7]. Hydrocortisone is the preferred type of steroid in the treatment of CAH treatment as it is a weak steroid. Good adherence is required for good outcomes. Undertreatment, overtreatment, and non-adherence may result in failure to thrive, suppressed bone development, reduced final height, and virilisation [8,10].

In previous studies conducted before the COVID-19 pandemic on pediatric patients with chronic diseases, 65-90% of patients did not adhere to treatment, with an average adherence rate of approximately 50% [11]. In contrast, this study found high adherence rates among patients with CAH both before and after the onset of the pandemic.

Many factors have affected medication adherence in the paediatric population during the COVID-19 pandemic, including individual and family characteristics, as well as community and healthcare system factors. On an individual level, changes to daily routines may affect adherence by increasing forgetfulness; however, increasing parental responsibilities, including home-schooling and home-working, may have disrupted child treatment adherence. Conversely, increased caregiver supervision may increase medication adherence among children [11]. In this study, the COVID-19 pandemic affected treatment adherence by disrupting medication supply and accessibility; nevertheless, forgetfulness was the most common reason for not taking the prescribed medication during the pandemic. Few studies have examined treatment adherence among children and adolescents with CAH. A previous study reported higher adherence rates among children and adolescents than among adults, most probably owing to parental supervision, and a non-adherence rate of 20% among children [12]. The present study's findings are consistent with those of previous studies done before the COVID-19 pandemic.

Patients with CAH require regular follow-up visits at the clinic. Patients aged ≤18 months require appointments every three months. Patients aged ≥18 months require appointments every three to six months with regular laboratory tests to evaluate serum electrolyte and plasma hormone levels and to make medication adjustments, as required. Outcomes associated with undertreatment can be monitored during these appointments, helping prevent failure to thrive, blood pressure abnormalities, and peripheral precocious puberty, leading to the early closure of the epiphyses [7,8]. This study included patients who attended the clinic regularly and received continuous care and information.

Paediatric treatment adherence is determined by many factors, including caregiver education level. Specifically, higher educational attainment of the parents is associated with higher treatment adherence rates of the children [6]. A study of children with chronic kidney disease found that higher maternal education levels were associated with higher child treatment compliance rates [13]. This finding is consistent with that of the present study. Medication characteristics such as regimen complexity, taste, and adverse effects may affect adherence. Relationships between patients and their care providers may affect adherence, especially among young children [6].

Limitations

This study has some limitations. The sample size was small because of the rarity of the disease. This study used self-reporting for older children (aged over 10 years) and parental reporting for younger children, which may have introduced respondent bias. Other limitations include the limited geographic area covered (one clinic in one city) and loss-to-follow-up during the COVID-19 pandemic, either due to the pandemic or due to lack of access to technology for virtual clinic follow-up. Future research should evaluate the adherence of patients with CAH in several different cities in Saudi Arabia and should also evaluate the effect of virtual clinics on patients with other chronic diseases.

## Conclusions

The COVID-19 pandemic introduced physicians to the value of virtual follow-ups as a convenient alternative to hospital visits for stable patients with chronic diseases. The results of this study suggest that this approach helps maintain high adherence rates. Virtual clinics are a suitable model of care for the follow-up of patients with chronic diseases, even under non-epidemic conditions.

## References

[1] El-Maouche D, Arlt W, Merke DP (2017). Congenital adrenal hyperplasia. Lancet.

[2] Alfadhel M, Al Othaim A, Al Saif S (2017). Expanded newborn screening program in Saudi Arabia: incidence of screened disorders. J Paediatr Child Health.

[3] Mohamed S, El-Kholy S, Al-Juryyan N, Al-Nemri AM, Abu-Amero KK (2015). A CYP21A2 gene mutation in patients with congenital adrenal hyperplasia. Molecular genetics report from Saudi Arabia. Saudi Med J.

[4] Yang W, Cao Q, Qin L (2020). Clinical characteristics and imaging manifestations of the 2019 novel coronavirus disease (COVID-19):A multi-center study in Wenzhou city, Zhejiang, China. J Infect.

[5] Alshehri LM, Al Agha AE (2021). Impact of Covid-19 lockdown on the unhealthy dietary habits and physical activity of children and adolescents living in the Kingdom of Saudi Arabia. Ann Med Health Sci Res.

[6] Dawson LA (2019). What factors affect adherence to medicines?. Arch Dis Child Educ Pract Ed.

[7] Speiser PW, Arlt W, Auchus RJ (2018). Congenital adrenal hyperplasia due to steroid 21-hydroxylase deficiency: An Endocrine Society clinical practice guideline. J Clin Endocrinol Metab.

[8] Wieacker I, Peter M, Borucki K, Empting S, Roehl FW, Mohnike K (2015). Therapy monitoring in congenital adrenal hyperplasia by dried blood samples. J Pediatr Endocrinol Metab.

[9] Alshareef R, Al Zahrani A, Alzahrani A, Ghandoura L (2020). Impact of the COVID-19 lockdown on diabetes patients in Jeddah, Saudi Arabia. Diabetes Metab Syndr.

[10] Alsalman AA, Aldossari MR, Alomani ZD, Alkhunaizi SI, Aljardah ZA, Almousa FA, Kalalah ZA (2022). Impact of coronavirus disease lockdown on children with type 1 diabetes mellitus in Al-Khobar, Saudi Arabia. Cureus.

[11] Plevinsky JM, Young MA, Carmody JK (2020). The impact of COVID-19 on pediatric adherence and self-management. J Pediatr Psychol.

[12] Jenkins-Jones S, Parviainen L, Porter J (2018). Poor compliance and increased mortality, depression and healthcare costs in patients with congenital adrenal hyperplasia. Eur J Endocrinol.

[13] Ramay BM, Cerón A, Méndez-Alburez LP, Lou-Meda R (2017). Factors associated to acceptable treatment adherence among children with chronic kidney disease in Guatemala. PLoS One.

